# Comparison of Random Forest and Stepwise Regression for Variable Selection Using Low Prevalence Predictors: A case Study in Paediatric Sepsis

**DOI:** 10.1007/s10995-025-04038-1

**Published:** 2025-01-15

**Authors:** Patricia Gilholm, Paula Lister, Adam Irwin, Amanda Harley, Sainath Raman, Luregn J Schlapbach, Kristen S Gibbons

**Affiliations:** 1 Children’s Intensive Care Research Program, Child Health Research Centre, The University of Queensland, Brisbane, QLD Australia; 2 Paediatric Critical Care Unit, Sunshine Coast University Hospital, Birtinya, QLD Australia; 3 School of Medicine, Griffith University, Nathan, QLD Australia; 4 UQ Centre for Clinical Research, The University of Queensland, Brisbane, QLD Australia; 5 Queensland Children’s Hospital, Brisbane, QLD Australia; 6 Department of Intensive Care and Neonatology, and Children’s Research Center, University Children’s Hospital Zurich, Zurich, Switzerland

**Keywords:** Low Prevalence Predictors, Variable Selection, Random Forest, Stepwise Logistic Regression, Paediatric sepsis

## Abstract

**Introduction:**

Variable selection is a common technique to identify the most predictive variables from a pool of candidate predictors. Low prevalence predictors (LPPs) are frequently found in clinical data, yet few studies have explored their impact on model performance during variable selection. This study compared the Random Forest (RF) algorithm and stepwise regression (SWR) for variable selection using data from a paediatric sepsis screening tool, where 18 out of 32 predictors had a prevalence < 10%.

**Methods:**

Variable selection using RF was compared to forward and backward SWR. Model performance was evaluated using the area under the receiver operating characteristic curve (AUC), and the variables retained. Additionally, a simulation study assessed how increasing the prevalence of the predictors impacted the variable selection results.

**Results:**

The best fitting RF and SWR models retained were 22, and 17 predictors, respectively, with 14 and 10 predictors having a prevalence < 10%. Both the RF and SWR models had similar predictive performance (RF: AUC [95% Confidence Interval] 0.79 [0.77, 0.81], LR: 0.80 [0.78, 0.82]). The simulation study revealed differences for both RF and SWR models in variable importance rankings and predictor selection with increasing prevalence thresholds, particularly for moderately and strongly associated predictors.

**Discussion:**

The RF algorithm retained a number of very low prevalence predictors compared to SWR. However, the predictive performance of both models were comparable, demonstrating that when applied correctly and the number of candidate predictors is small, both methods are suitable for variable selection when using low prevalence predictors.

**Supplementary Information:**

The online version contains supplementary material available at 10.1007/s10995-025-04038-1.

## Significance

What is Already Known on this Subject?

What this Study adds?

Low prevalence predictors (LPPs) are common in clinical data and require special consideration during the variable selection process. Random Forest (RF) is an efficient and increasingly popular algorithm for variable selection. Numerous approaches have been developed using RF for variable selection, which take advantage of the variable importance measures produced by the algorithm.

This study evaluates the performance of RF for variable selection when using LPPs. The RF and stepwise regression had comparable predictive performance; however, the RF was able to account for complex interactions and was less sensitive to the prevalence of the variables.

**Supplementary Information:**

The online version contains supplementary material available at 10.1007/s10995-025-04038-1.

## Introduction

Variable selection is a modelling technique in which the most predictive variables are selected from a pool of candidate predictors, with the goal of obtaining the smallest set that can still achieve good predictive performance (George, 2000). Common variable selection techniques such as bivariate analysis (Sun et al., 1996) and forward and backward stepwise regression (SWR) (Harrel, 2001) have been shown to introduce systematic biases to the estimated parameters (Heinze & Dunkler, 2017). An alternative approach to variable selection, which may mitigate the problems identified in the more common techniques, is the Random Forest (RF) algorithm.

The Random Forest (Breiman, 2001) is an efficient algorithm used for modelling high-dimensional data and correlated predictors (Boulesteix et al., 2012). Numerous procedures have been developed and explored using RF for variable selection (Diaz-Uriarte & Alvarez de Andres, 2006; Fox et al., 2017; Genuer et al., 2010) and are gaining prominence in ecology (Fox et al., 2017), bioinformatics (Boulesteix et al., 2012), and clinical research (Lamping et al., 2018; Sanchez-Pinto et al., 2018). However, the impact of these methods when applied to data containing low prevalence predictors (LPPs) has yet to be explored.

Binary low-prevalence predictors (LPPs) are common in medical research, particularly when data is extracted from electronic health records (EHRs). Low prevalence predictors often arise in child and maternal health studies, as well as other areas of medical research, due to the nature of certain medical conditions or risk factors that have low incidence rates (e.g., maternal smoking, history of gestational diabetes in many communities) (Maher et al., 2024). Using LPPs in regression modelling can lead to convergence problems resulting in large and unstable parameter estimates and inflated standard errors (Steyerberg, 2019). These issues arise because traditional variable selection methods, such as SWR, may struggle to handle variables that occur rarely in the data while still retaining meaningful predictive power (Steyerberg, 2019). Despite these challenges, LPPs are rarely considered or addressed during variable selection, with previous research predominantly focused on the impact of low prevalence outcome variables and the strategies to address them (Boulesteix et al., 2012; Harrel, 2001). Two previous simulation studies that specifically addressed the use of LPPs demonstrated stable performance only when a large sample size was used or when LPPs had a very strong association with the outcome (Ogundimu et al., 2016; Schummers et al., 2016).

This study investigated the use of the RF algorithm, compared with stepwise regression for variable selection when modelling LPPs. For this comparison we used data from a paediatric sepsis screening tool that contained a high proportion of LPPs. A simulation study was also undertaken to determine the impact of the prevalence of the predictors on the variables selected and the predictive performance of the models.

## Methods

### Data

Data from 3473 children, who were screened for sepsis in the ED between 2018 and 2019 were used in this comparison; this cohort has been reported on previously (Harley et al., 2021). The outcome variable used in this study was *sepsis*, diagnosed in the ED; 523 (15.1%) children were classified as having *sepsis*. Ethical approval for use of the data in this study was granted by the Children’s Health Queensland Human Research Ethics Committee including waiver of individual consent (HREC/18/QRCH/167).

Predictors were taken from the screening tool, which contained 32 criteria: (1) nine Sepsis Indicators, (2) six Sepsis Risk Factors, (3) eight Severe Illness Features and (4) nine Moderate Illness Features (Online Resource 1 eTable 1). Eighteen of the 32 criteria were LPPs, defined as predictors with a prevalence < 10% (Online Resource 1 eTable 1). Of these, 12 criteria had ≤ 5% prevalence.

### Random Forest Variable Selection Procedure

Variable selection was undertaken using the permutation variable importance metrics (Goldstein et al., 2011), following the procedures outlined in Behnamian et al. (2017) and Genuer et al. (2010) (Online Resource 1 Supplementary Methods). Briefly, 25 RFs, each consisting of 10,000 trees to ensure stability of the importance metrics, were estimated and the average variable importance for each predictor were calculated and ranked (Behnamian et al., 2017). After removal of predictors with negative variable importance, a series of RFs were constructed, sequentially adding an additional ranked predictor for each RF. The area under the receiver operating characteristic curves (AUCs) and 95% confidence intervals (CIs, computed with stratified bootstrap replicates) for each model were calculated through 10-fold cross-validation and compared to an RF with all predictors using DeLong’s method (DeLong et al., 1988).

The *mtry* hyperparameter of the RF defines the number of candidate predictors selected for each split of a tree (Diaz-Uriarte & Alvarez de Andres, 2006). To investigate the impact of the *mtry* hyperparameter when modelling LPPs, the RF variable selection procedure was performed using three different values for *mtry*, corresponding to a small number (*mtry =* 3), the default (*mtry=*√*p* = 6) and a large number (*mtry =* 16). The RF models were constructed using the randomForest package in R (Liaw & Wiener, 2002).

### Stepwise Regression Variable Selection

Both forward and backward stepwise regression techniques, using the Akaike Information Criterion (AIC) as the criterion for predictor selection, were used as a comparison (Harrel, 2001). The backward SWR model using this data has been developed and reported on previously (Gilholm et al., 2023). The forward SWR model, backward SWR model and the full model containing all predictors were validated using 10-fold cross-validation and the AUC and 95% CI were calculated on the hold-out test folds and compared across models. The strength of each predictor was assessed by the odds ratio and corresponding 95% confidence intervals (CIs).

All modelling was performed in R (version 4.0.2) (R Core Team, 2020) and code is available online (https://github.com/TrishGilholm/RFvsLR_low_prevalence_predictors).

### Simulation Study Methods

A simulation study was performed to investigate the impact that increasing the prevalence of the predictors has on the predictive performance of the models and the variables retained during variable selection. Data were simulated at four different prevalence thresholds; the prevalence observed in the data, and a minimum prevalence of 10%, 20%, and 30% (Online Resource 1 Supplementary Methods).

Once the 4000 datasets were generated (1000 datasets for each of the four prevalence thresholds), an RF model using 10-fold cross validation was fit to each dataset and the permutation variable importance and AUC were recorded. To summarise the importance of each predictor, the variable importance measures were ranked within each simulated dataset from one (highest variable importance) to 32 (lowest variable importance). The distribution of the rankings for the simulated datasets within a prevalence threshold were calculated and compared across thresholds. The mean and standard deviation of the AUCs for each prevalence threshold were also compared.

For the SWR simulation, a logistic regression model using SWR was fit to the 4000 datasets. The number of times a variable was retained across the 1000 datasets within each prevalence threshold was calculated and compared. The AUC for each model was calculated using 10-fold cross-validation and the mean and standard deviation of the AUCs for each prevalence threshold were compared.

To simplify the simulations, only one RF *mtry* specification (either 3, 6 or 16) and one SWR procedure (either forward or backward) were used, corresponding to the models with the best predictive performance (based on AUC).

## Results

### Random Forest Variable Selection

**Fig. 1:**
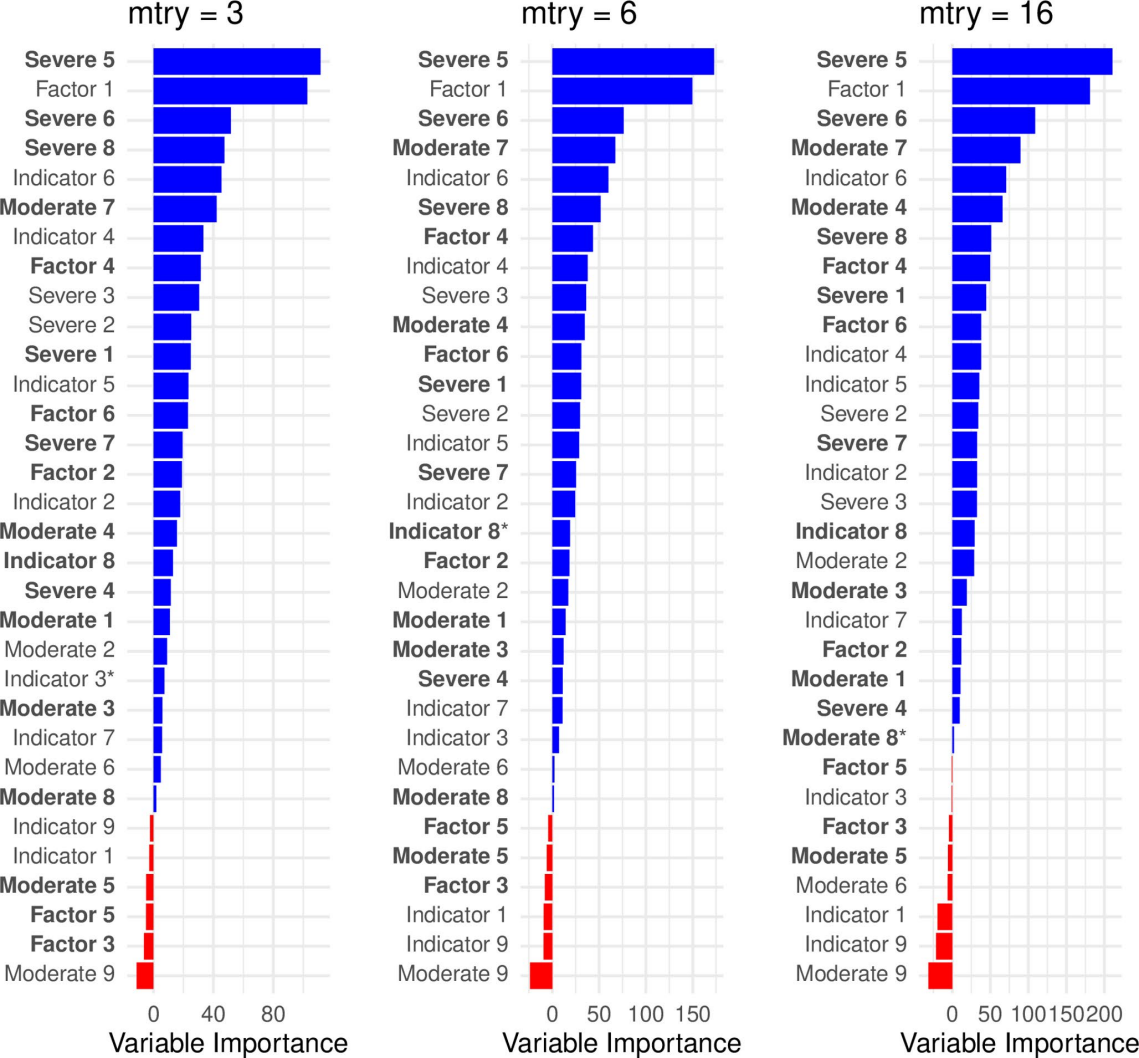
Average variable importance across the 25 iterations of the Random Forest for each of the *mtry* specifications. Low prevalence predictors (prevalence < 10%) are in bold. The variables retained during the variable selection procedure include all variables ranked above and including the one marked with an asterisk

The average variable importance across 25 RFs at the three specifications for *mtry* are displayed in Fig. 1 (Online Resource 1 eTable 2). Six of the top ten ranked predictors for *mtry* = 3 and *mtry* = 6, and eight of the top ten ranked predictors for *mtry* = 16, had a prevalence < 10%. The same six predictors returned negative variable importance values for *mtry* = 3 and *mtry* = 6. An additional two predictors returned negative importance values for *mtry* = 16. The performance of the models were similar between *mtry* = 3 and *mtry* = 6 (*mtry* = 3: AUC (95% CI) of mean predictions 0.80 (0.78, 0.82); *mtry* = 6: AUC (95% CI) of mean predictions 0.79 (0.77, 0.81)). The model with *mtry* = 16 had a lower AUC (AUC (95% CI) of mean predictions 0.78 (0.76, 0.80)).

For *mtry* = 3 (Table 1), the most parsimonious model with an AUC that did not differ significantly from the full model contained 22 predictors, of which 14 had a prevalence < 10% (Fig. 1). The variables selected consisted of six sepsis indicators, four sepsis risk factors, all eight severe illness features and four moderate illness features. For *mtry* = 6 (Online Resource 1 eTable 3), the most parsimonious model contained 17 predictors (10 with prevalence < 10%, Fig. 1) and for *mtry* = 16 (Online Resource 1 eTable 4), the model containing all predictors with positive variable importance values performed worse than the full model containing all predictors (Fig. 1).

**Table 1:** The AUC and 95% CIs, and *p-*value for DeLong’s test for two correlated ROC curves for each Random Forest variable selection model for *mtry* = 3

Number of ranked features	AUC (95% CI)	*p*-value for DeLong’s test for two correlated ROC curves
3	0.66 (0.64,0.68)	< 0.001
4	0.66 (0.64, 0.68)	< 0.001
5	0.69 (0.66, 0.71)	< 0.001
6	0.69 (0.67, 0.71)	< 0.001
7	0.70 (0.68, 0.72)	< 0.001
8	0.72 (0.69, 0.74)	< 0.001
9	0.72 (0.70, 0.74)	< 0.001
10	0.72 (0.70, 0.74)	< 0.001
11	0.75 (0.72, 0.77)	< 0.001
12	0.76 (0.74, 0.78)	< 0.001
13	0.76 (0.73, 0.78)	< 0.001
14	0.76 (0.74, 0.79)	< 0.001
15	0.76 (0.74, 0.79)	< 0.001
16	0.77 (0.75, 0.80)	< 0.001
17	0.78 (0.76, 0.80)	0.003
18	0.78 (0.76, 0.81)	0.019
19	0.78 (0.76, 0.80)	0.002
20	0.78 (0.76, 0.80)	0.004
21^a^	0.79 (0.76, 0.81)	0.036
22	0.79 (0.77, 0.81)	0.211
23	0.80 (0.77, 0.82)	0.622
24	0.80 (0.78, 0.82)	0.898
25	0.80 (0.79, 0.82)	0.671
26	0.79 (0.77, 0.82)	0.369

AUC: Area under the receiver operating characteristic Curve; CI Confidence interval, ROC receiver operating characteristic. ^a^ The most parsimonious model with an AUC that did not differ significantly from the full model

### Stepwise Regression Variable Selection

The variables selected to be retained in the backward SWR model consisted of three sepsis indicators, three sepsis risk factors, all eight severe illness features, and three moderate illness features, for a total of 17 predictors retained (Table 2). Two of the predictors retained in the model were not statistically significant, specifically, hypotension (Severe 4), and capillary refill time ≥ 3 seconds (Moderate 3). Both of these predictors had very low prevalence (Severe 4: 1%, Moderate 3: 4%). Overall, 10 out of the 17 predictors selected had a prevalence < 10%.

**Table 2:** Odds ratios (OR), 95% confidence intervals (CI) and AUC for the backward model, forward model, and full model

Coefficient	Backward Model		Forward Model		Full Model	
	OR	95% CI	OR	95% CI	OR	95% CI
Sepsis Indicators						
1. Parental concern	-	-	-	-	1.03	0.82, 1.28
2. Healthcare worker concern	1.45	1.16, 1.81	1.46	1.17, 1.82	1.44	1.15, 1.80
3. History of fever or hypothermia	-	-	-	-	0.96	0.76, 1.20
4. Looks sick	1.48	1.17, 1.88	1.50	1.19, 1.90	1.49	1.17, 1.89
5. Altered behaviour or reduced level of consciousness	1.36	1.02, 1.80	1.38	1.03, 1.83	1.35	1.01, 1.80
6. Total CEWT score of 4 or more	-	-	-	-	1.12	0.87, 1.44
7. Re-presentation within 48 h	-	-	-	-	1.19	0.76, 1.81
**8. Unexplained pain/restlessness**	-	-	-	-	1.11	0.82, 1.49
9. Deterioration during current illness	-	-	-	-	0.98	0.71, 1.33
Sepsis Risk Factors						
1. Age less than 3 months	4.61	3.48, 6.10	4.71	3.56, 6.23	4.60	3.46, 6.13
**2. Indwelling medical device**	-	-	-	-	1.18	0.54, 2.43
**3. Aboriginal and Torres Strait Islander/Pacific Islander/Maori**	-	-	-	-	1.04	0.63, 1.67
**4. Immunocompromised/ asplenia/neutropenia/unimmunised**	3.12	1.88, 5.05	3.05	1.84, 4.93	3.02	1.80, 4.96
**5. Recent trauma or surgery/invasive procedure/wound within the last 6 weeks**	-	-	-	-	1.54	0.79, 2.84
**6. Chronic disease or congenital disorder**	1.92	1.21, 2.99	1.97	1.25, 3.05	1.84	1.13, 2.95
Severe Illness Features						
**1. Need oxygen to keep oxygen saturation ≥ 92%**	1.93	1.28, 2.87	1.93	1.28, 2.86	1.87	1.24, 2.80
2. Severe respiratory distress/tachypnoea/apnoea (CEWT respiratory score 3)	1.74	1.26, 2.39	1.70	1.23, 2.33	1.67	1.20, 2.31
3. Severe tachycardia or bradycardia (CEWT heart rate score 3)	2.03	1.54, 2.68	2.01	1.52, 2.64	1.94	1.44,2.60
**4. Hypotension (CEWT blood pressure score > = 2)**	2.13	0.81, 5.38	-	-	2.13	0.81, 5.41
**5. Lactate ≥ 2mmol/L**	6.36	4.76, 8.51	6.27	4.70, 8.37	6.30	4.70, 8.47
**6. Altered AVPU**	2.00	1.29, 3.09	2.03	1.31, 3.12	2.00	1.28, 3.10
**7. Non-blanching rash**	2.57	1.57, 4.13	2.57	1.57, 4.11	2.57	1.57, 4.14
**8. Hypothermia (CEWT temperature score 2)**	7.05	2.78, 17.8	6.82	2.69, 17.2	6.89	2.71, 17.5
Moderate Illness Features						
**1. Moderate respiratory distress/tachypnoea (CEWT respiratory score 2)**	-	-	-	-	0.83	0.50, 1.33
2. Moderate tachycardia (CEWT heart rate score 2)	-	-	-	-	1.12	0.76, 1.63
**3. Capillary refill ≥ 3 s**	1.55	0.92, 2.51	-	-	1.68	0.97, 2.81
**4. Unexplained pain or restlessness**	-	-	-	-	0.82	0.46, 1.38
**5. Low blood glucose level**	-	-	-	-	1.37	0.39, 3.70
6. Pale or flushed/mottled	-	-	-	-	0.89	0.58, 1.32
**7. Cold extremities**	2.53	1.42, 4.31	2.72	1.55, 4.58	2.58	1.43, 4.49
**8. Reduced urine output**	-	-	-	-	0.87	0.53, 1.36
9. Parental/healthcare worker concern	1.37	1.02, 1.84	1.38	1.03, 1.85	1.41	1.03, 1.92
*AUC*	*0.80*	*0.78*,* 0.82*	*0.80*	*0.78*,* 0.82*	*0.80*	*0.78*,* 0.82*

CEWT: Children’s Early Warning Tool; AVPU: Alert, Voice, Pain, Unresponsive scale; OR: Odds Ratio; CI: Confidence Interval; AUC: Area Under the receiver operating characteristic Curve. Low prevalence predictors (prevalence < 10%) are in bold

The forward SWR model retained the same predictors as the backward SWR model, except the two predictors that were not statistically significant that were retained in the backward SWR model were not selected in the forward SWR model. The forward, backward and full model had equivalent predictive performance (AUC 0.80, 95% CI 0.78, 0.82).

### Simulation Study Results

**Fig. 2:**
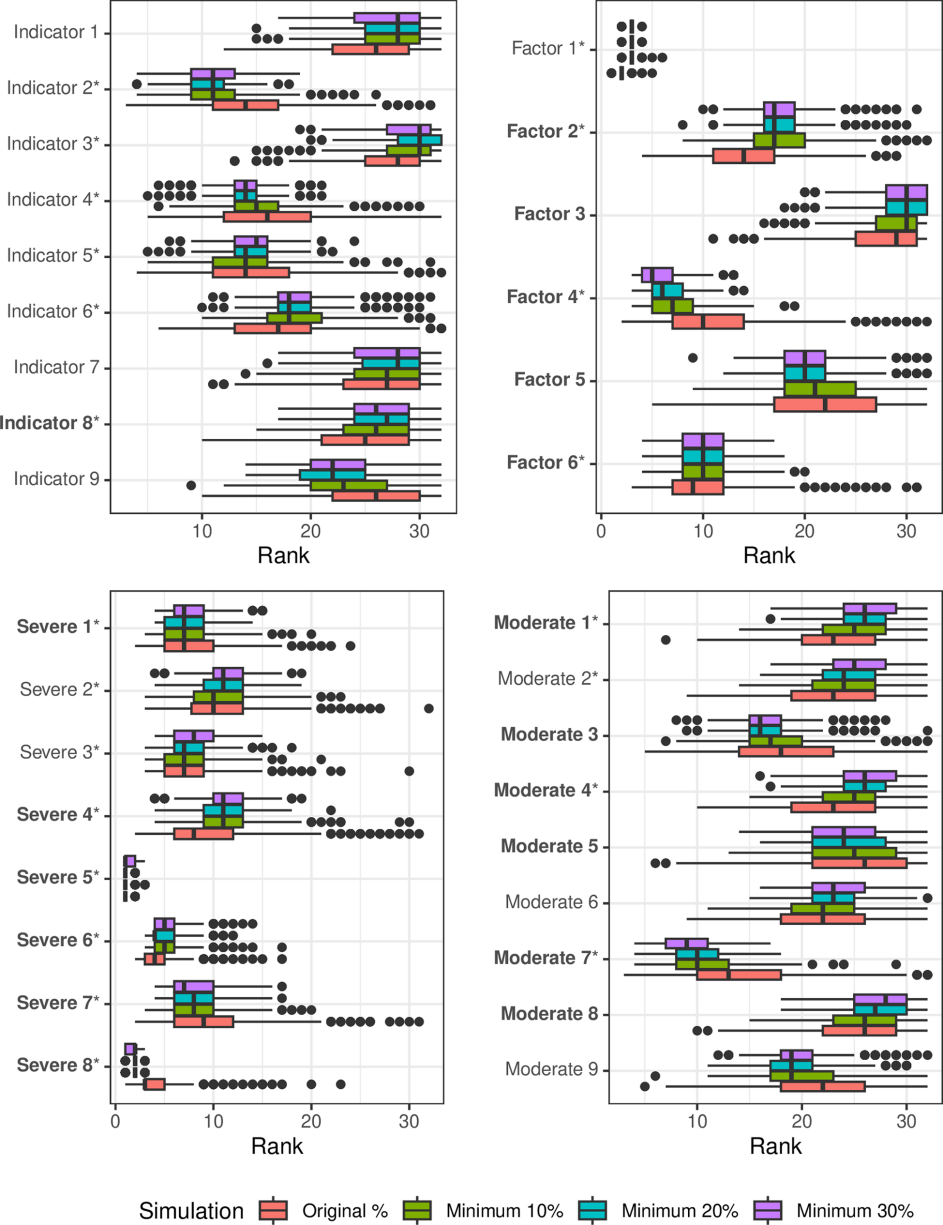
Distribution of the variable importance rankings for each predictor within each prevalence threshold. Variables are ranked from one (highest variable importance) to 32 (lowest variable importance). Bold labels indicate low prevalence predictors. Variables marked with an asterisk correspond to the retained predictors in the mtry = 3 Random Forest model

For the RF simulations (constructed using *mtry = 3*), the distribution of the variable importance rankings were similar for most predictors across the 10–30% prevalence thresholds (Fig. 2), however, there were differences observed between the original prevalence threshold and the higher prevalence thresholds, particularly for the LPPs. For most LPPs that had a strong association with the outcome (i.e., ranked in the top 10 in the original RF analysis), there was less variability in the rankings and a shift upwards in the distribution of the rankings (e.g., Severe 8, Moderate 7, Factor 4) for the higher prevalence thresholds compared to the original prevalence. In contrast, for LPPs that had a moderate association with the outcome (ranked 11th-22nd in the original RF analysis), there was less variability, but with a shift downwards in the distribution of the rankings (e.g., Factor 2, Moderate 4, Severe 4) for the higher prevalence thresholds compared to the original prevalence. For all variables with weak associations with the outcome (variables not retained in the original RF analysis), there was more variability in the rankings for the original prevalence threshold (e.g., Moderate 3, Moderate 8, Moderate 5), but the location of the distributions remained the same at the higher prevalences. Overall, the predictive performance of the models increased with increasing prevalence sizes (Mean AUC (SD) Original = 0.79 (0.01), 10% = 0.82 (0.01), 20% = 0.85 (0.01), 30% = 0.86 (0.01)).

**Fig. 3:**
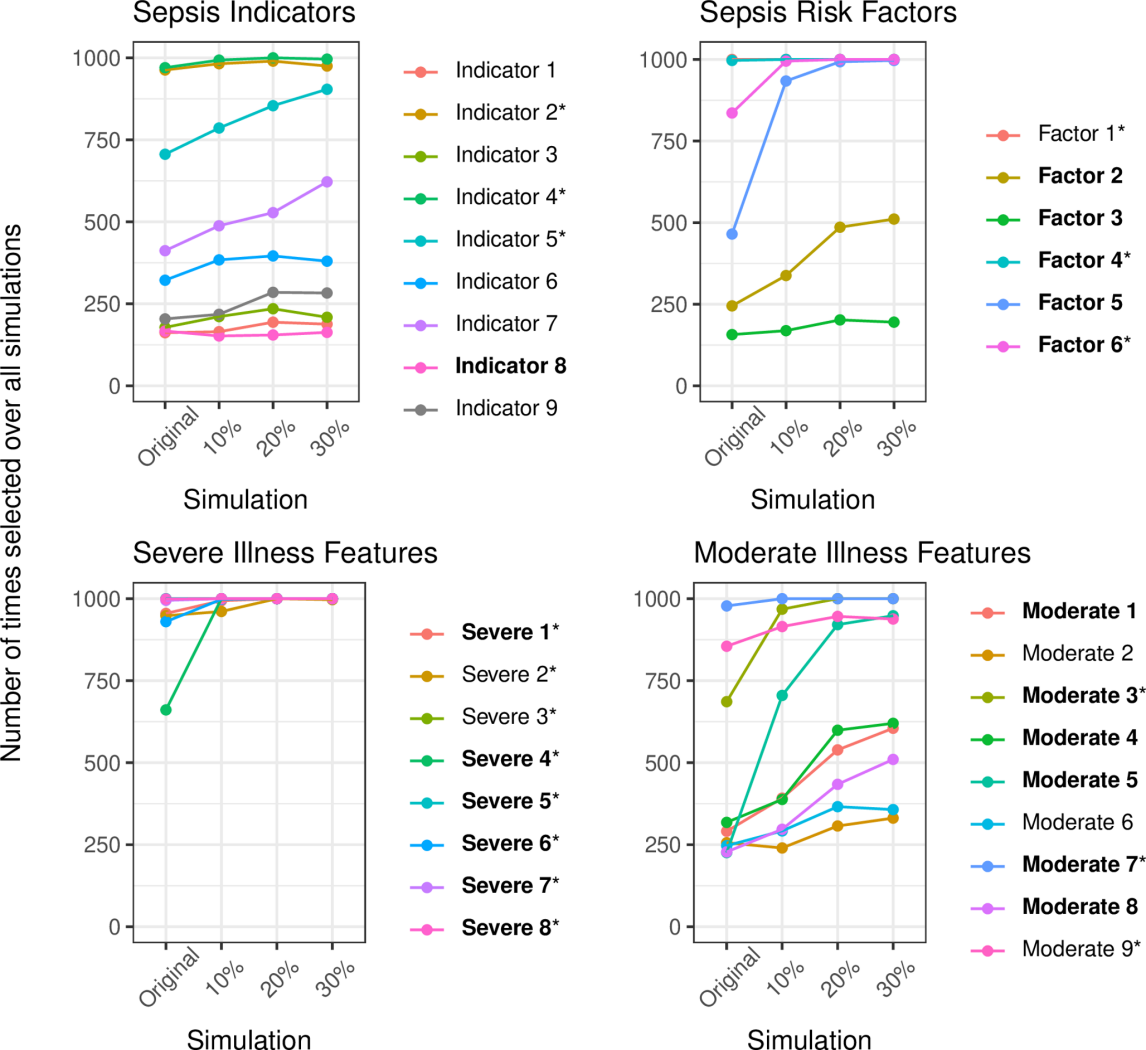
Number of times out of 1000 simulations each variable was retained in the backward SWR procedure, for each of the four prevalence thresholds. Bold labels indicate low prevalence variables. Variables marked with an asterisk correspond to the retained predictors in the backward stepwise regression model

For the SWR simulations (constructed using backward SWR), the predictors that were retained in the model were retained for the majority of the simulations across all prevalence thresholds (Fig. 3). Two of the predictors (recent trauma or surgery [Factor 5], and low blood glucose level [Moderate 5]) were not retained in the original modelling but showed an increase in the number of times they were selected when the prevalence of the predictors increased. Each of these predictors had a very low prevalence of ≤ 4%. Overall, the predictive performance of the models increased with increasing prevalence sizes (Mean AUC (SD) Original = 0.81 (0.01), 10% = 0.84 (0.01), 20% = 0.86 (0.01), 30% = 0.88 (0.01)).

## Discussion

The purpose of this study was to compare the performance of RF and SWR variable selection methods for data containing many LPPs. Although previous studies have compared the performance of RF and SWR for clinical prediction in general (Christodoulou et al., 2019), these studies have not focused on the impacts of LPPs. Using the screening criteria from a paediatric sepsis screening tool, we have shown that although both RF and SWR approaches had comparable predictive performance, the RF algorithm was less sensitive to the prevalence of the predictors and retained more LPPs, compared to the SWR models.

When comparing the variables that were retained by each procedure, several predictors were retained using RF that were not selected in the SWR models, including four LPPs. Previous research (Fox et al., 2017) has shown that to obtain predicted probabilities comparable to those produced by an RF using all candidate predictors required a larger subset of predictors identified through variable selection. Our study supports this finding, as 50–70% of the ranked variables needed to be included during the variable selection process, for both mtry = 3 and mtry = 6, to achieve model performance comparable to the full model using all predictors. For mtry = 16, all sub-models of the ranked features performed worse than the full model with all predictors. One predictor selected by the SWR models, specifically the combined parental and healthcare worker concern (Moderate 9), consistently ranked lowest in variable importance across all RF models. As both parental concern and healthcare worker concern were also included as separate predictors (Indicators 1 and 2), thanks to the ability of RF to handle complex interactions (Boulesteix et al., 2012), it identified that healthcare worker concern alone was predictive, while parental concern contributed little to the model. This demonstrates RF’s strength in handling correlated predictors.

With the RF variable selection procedure, the *mtry* parameter influenced the variable importance rankings and the overall predictive performance of the model. In this application, a smaller value for *mtry* resulted in better predictive performance. Smaller values for *mtry* mean that LPPs with a moderate association have more opportunity to contribute to the prediction and are not overshadowed by strong predictors with larger prevalence (Fox et al., 2017). However, the simulation study revealed that predictors with a weaker association had more variability in importance rankings compared to predictors with a strong association. This may be because the use of a small value for *mtry* led to more importance being given to uninformative predictors, resulting in instability in variable importance (Boulesteix et al., 2012). These results demonstrate the importance of tuning the *mtry* parameter when performing variable selection using LPPs.

For stepwise regression modelling, although the forward and backward SWR models were largely similar and had identical predictive performance, the backward SWR model included two LPPs which did not have a significant association with the outcome. In general, backward SWR models are preferred over forward SWR as the backward SWR starts with the full model and retains the dependency structure between the variables (Harrel, 2001). This appears to be important when modelling LPPs, as the two additional variables that were retained had very low prevalence, but substantial effect sizes, which still contributed to the overall model performance. The SWR simulation study identified a further two predictors that were retained when the prevalence of the predictors increased, showing that SWR techniques may be sensitive to the prevalence of predictors that have a moderate association with the outcome. These results also highlight the perils of using *p*-values as the stopping criteria for variable selection (Harrel, 2001), as the non-significant LPPs would not have been retained if that criterion was used. As one of the non-significant LPPs retained in the backward elimination procedure was hypotension (a key indicator of septic shock), it would make little clinical sense to remove this criterion from a sepsis screening tool, providing further support that statistical significance does not always equate to practical or clinical significance.

The overall predictive performance of the backward SWR model was similar to that produced by the RF algorithm when a small *mtry* was specified. Previous studies have shown similar results when a relatively small number of predictors are used (Sanchez Fernandez et al., 2018). Improved performance of the RF algorithm may only be observed in high-dimensional setting when hundreds or thousands of predictors are used (Fox et al., 2017). Of note, the computational resources needed for RF were much more substantial than SWR. We utilised high-performance computing resources to perform the sequential variable selection procedure in parallel, and this resource requirement should be considered when comparing methods.

### Limitations

The data used in this study was imbalanced and RFs are known to be sensitive to class imbalances. Sampling strategies are often used to balance the response classes (Fox et al., 2017), which may have improved the RFs performance, however, as our goal was to investigate the impact of LPPs this would likely lead to an inflation in the prevalence of the predictors, so was not performed. Typically, the value for *mtry* and other hyperparameters would be optimised using a grid or random search procedure. For this study, hyperparameter optimisation was not performed, and instead three values (representing a small *mtry*, the default, and a large *mtry*) were selected a priori, to explore the potential impact that the *mtry* parameter may have when modelling LPPs. Although our selection of *mtry* values may not have included the most optimal value, our process reflects the standard procedure of a hyperparameter grid search, whereby three or four candidate values for each hyperparameter are explored. Finally, this study only compared stepwise regression and Random Forest procedures for variable selection. While many other methods exist, we chose to focus on an in-depth comparison between a newer technique (RF) and traditional variable selection procedures (SWR), rather than an exhaustive comparison of all available methods.

## Conclusions

Low prevalence predictors are a common occurrence in clinical applications, but the methodological challenges associated with their use are often not considered in variable selection procedures. This study has shown that the RF algorithm may be less sensitive to LPPs and may be a more practical alternative for variable selection in high-dimensional settings.

## Electronic Supplementary Material

Below is the link to the electronic supplementary material.

Supplementary Material 1

## Data Availability

Data inquiries should be addressed to the corresponding author. The data are not publicly available due to privacy and ethical restrictions. Approval of data release will be governed by the Queensland Sepsis Collaborative and The University of Queensland.

## Code Availability

The R codes used for the modelling in this paper are available online (https://github.com/TrishGilholm/RFvsLR_low_prevalence_predictors).
