# Supplementary material for: Comparison of Random Forest and Stepwise Regression for Variable Selection Using Low Prevalence Predictors: A case Study in Paediatric Sepsis
Source: Matern Child Health J. 2025 Jan 15;29(5):604–13. doi: 10.1007/s10995-025-04038-1 (PMC12098530; doi:10.1007/s10995-025-04038-1)
Supplement: Supplementary file 1 — Supplementary Material 1 [file 10995_2025_4038_MOESM1_ESM.docx]

Supplementary Material for:

Comparison of Random Forest and Stepwise Regression for variable selection using low prevalence predictors: A case study in paediatric sepsis.

Maternal and Child Health Journal

Patricia Gilholm^1^, Paula Lister, Adam Irwin, Amanda Harley, Sainath Raman, Luregn J Schlapbach, Kristen S Gibbons

^1^Corresponding author: Children’s Intensive Care Research Program, Child Health Research Centre, The University of Queensland, email: p.gilholm@uq.edu.au

Table of Contents

1. Supplementary Methods
2. eTable 1: List of variables used for variable selection
3. eTable 2: Average variable importance, OOB error and AUC across the 25 iterations for each specification of mtry.
4. eTable 3: OOB error, AUC, and p-value for DeLong’s test for two correlated ROC curves for each variable selection model for mtry =6.
5. eTable 4: OOB error, AUC, and p-value for DeLong’s test for two correlated ROC curves for each variable selection model for mtry =16.

Supplementary Methods

*Random Forest variable selection procedure*

The following steps were performed for variable selection:

(a) Twenty-five RFs, each consisting of ntree = 10,000 trees were estimated and the average variable importance and area under the receiver operating characteristic curve (AUC) across the 25 RFs were calculated to provide a stable estimate of the variable importance for each variable. ^1^

(b) The predictors were ranked according to their average variable importance and predictors with negative variable importance were removed. ^2^

(c) Following the procedure outlined in Genuer et al (2010), ^2^ using the remaining variables, *k=p-(mtry-1)* RFs were constructed, each containing *ntree* = 10,000, where model 1 was built using the *mtry* number of most important predictors, model 2 contained *mtry +1* most important predictors etc. until model *k* which contained all *p* predictors, where *p* is the total number of predictors with positive variable importance.

(d) The AUCs for each model were calculated using predictions on the test folds generated through 10-fold cross-validation. Delonge’s method^3^ was used to compare the receiver operating characteristic (ROC) curve from each sub-model to the ROC curve of the full model containing all predictors. The final model was selected as the model that was the most parsimonious with no significant difference in ROC curves to the full model with all predictors.

*Methods for data simulation*

The predictors for each simulated dataset were generated using the R package “bindata” ^4^ to generate artificial binary data. For each prevalence threshold, 1000 random datasets of n =3500 and p = 32 were generated. For each predictor, if the original prevalence observed in the data exceeded the minimum prevalence for each threshold, the predictor was simulated at the prevalence observed in the data. To generate the response variable, the predicted probabilities from the logistic regression model containing all predictors were extracted from each simulated dataset and then converted to a binary variable through a random draw from a Bernoulli distribution at the obtained predicted probability.

References:

1. Behnamian A, Millard K, Banks SN, White L, Richardson M, Pasher J. A Systematic Approach for Variable Selection With Random Forests: Achieving Stable Variable Importance Values. IEEE Geoscience and Remote Sensing Letters. 2017;14(11):1988-1992. doi:10.1109/lgrs.2017.2745049

2. Genuer R, Poggi J-M, Tuleau-Malot C. Variable selection using random forests. Pattern Recognition Letters. 2010;31(14):2225-2236. doi:10.1016/j.patrec.2010.03.014

3. DeLong ER, DeLong DM, Clarke-Pearson DL. Comparing the areas under two or more correlated receiver operating characteristic curves: a nonparametric approach. Biometrics. 1988:837-845.

4. Leisch F, Weingessel, A., & Hornik, K. On the generation of correlated binary data, Technical report, WU Vienna University of Economics and Business. 1998;

2. eTable 1: List of criteria outlined on the screening tool which were used for variable selection and the prevalence (%) of each criteria in the sample (N=3473).

| **Variable Type** | **Name** | **Description** | **Prevalence (%)** |
| --- | --- | --- | --- |
| Sepsis Indicators | Indicator 1 | Parental Concern | 55% |
|  | Indicator 2 | Healthcare worker concern | 34% |
|  | Indicator 3 | History of fever or hypothermia | 65% |
|  | Indicator 4 | Looks sick | 46% |
|  | Indicator 5 | Altered behaviour or reduced level of consciousness | 14% |
|  | Indicator 6 | Total CEWT score of 4 or more | 36% |
|  | Indicator 7 | Re-presentation within 48 hours | 13% |
|  | Indicator 8 | Unexplained pain/restlessness | 6% |
|  | Indicator 9 | Deterioration during current illness | 16% |
| Sepsis Risk Factors | Factor 1 | Age less than three months | 11% |
|  | Factor 2 | Indwelling medical device | 2% |
|  | Factor 3 | Aboriginal and Torres Strait Islander/Pacific Islander/Maori | 5% |
|  | Factor 4 | Immunocompromised/ asplenia/neutropenia/unimmunised | 3% |
|  | Factor 5 | Recent trauma or surgery/invasive procedure/wound with the last 6 weeks | 2% |
|  | Factor 6 | Chronic disease or congenital disorder | 4% |
| Severe Illness Features | Severe 1 | Need oxygen to keep oxygen saturation ≥ 92% | 6% |
|  | Severe 2 | Severe respiratory distress/ tachypnoea/ apnoea (CEWT repsitatory score 3) | 10% |
|  | Severe 3 | Severe tachycardia or bradycardia (CEWT heart rate score 3) | 15% |
|  | Severe 4 | Hypotension (CEWT blood pressure score ≥ 2) | 1% |
|  | Severe 5 | Lactate ≥ 2mmol/L | 9% |
|  | Severe 6 | Altered AVPU | 4% |
|  | Severe 7 | Non-blanching rash | 4% |
|  | Severe 8 | Hypothermia (CEWT temperature score 2) | 1% |
| Moderate Illness Features | Moderate 1 | Moderate respiratory distress/tachypnoea (CEWT respiratory score 2) | 8% |
|  | Moderate 2 | Moderate tachycardia (CEWT heart rate score 2) | 14% |
|  | Moderate 3 | Capillary refill ≥ 3 seconds | 4% |
|  | Moderate 4 | Unexplained pain or restlessness | 7% |
|  | Moderate 5 | Low blood glucose level | 1% |
|  | Moderate 6 | Pale or flushed/mottled | 12% |
|  | Moderate 7 | Cold extremities | 3% |
|  | Moderate 8 | Reduced urine output | 8% |
|  | Moderate 9 | Parental/healthcare worker concern | 20% |

CEWT Children’s Early Warning Tool; AVPU Alert, Verbal, Pain, Unresponsive scale

2. eTable 2: Average variable importance across the 25 iterations for each specification of *mtry*.

| *mtry* = 3 | | *mtry* = 6 | | *mtry* = 16 | |
| --- | --- | --- | --- | --- | --- |
| Variable | Importance | Variable | Importance | Variable | Importance |
| **Severe 5** | 111.56 | **Severe 5** | 172.81 | **Severe 5** | 210.52 |
| Factor 1 | 102.68 | Factor 1 | 149.54 | Factor 1 | 180.98 |
| **Severe 6** | 51.69 | **Severe 6** | 76.24 | **Severe 6** | 108.84 |
| **Severe 8** | 47.31 | **Moderate 7** | 67.24 | **Moderate 7** | 89.64 |
| Indicator 6 | 45.43 | Indicator 6 | 59.89 | Indicator 6 | 70.84 |
| **Moderate 7** | 42.19 | **Severe 8** | 51.56 | **Moderate 4** | 66.09 |
| Indicator 4 | 33.33 | **Factor 4** | 43.39 | **Severe 8** | 51.05 |
| **Factor 4** | 31.52 | Indicator 4 | 37.73 | **Factor 4** | 49.67 |
| Severe 3 | 30.51 | Severe 3 | 36.19 | **Severe 1** | 44.49 |
| Severe 2 | 25.17 | **Moderate 4** | 34.72 | **Factor 6** | 37.95 |
| **Severe 1** | 24.88 | **Factor 6** | 30.99 | Indicator 4 | 37.92 |
| Indicator 5 | 23.34 | **Severe 1** | 30.87 | Indicator 5 | 35.48 |
| **Factor 6** | 23.04 | Severe 2 | 29.60 | Severe 2 | 34.05 |
| **Severe 7** | 19.34 | Indicator 5 | 28.69 | **Severe 7** | 32.62 |
| **Factor 2** | 19.02 | **Severe 7** | 25.37 | Indicator 2 | 32.59 |
| Indicator 2 | 17.85 | Indicator 2 | 24.36 | Severe 3 | 32.28 |
| **Moderate 4** | 15.69 | **Indicator 8** | 18.95 | **Indicator 8** | 29.29 |
| **Indicator 8** | 12.97 | **Factor 2** | 18.27 | Moderate 2 | 28.62 |
| **Severe 4** | 11.49 | Moderate 2 | 17.11 | **Moderate 3** | 19.04 |
| **Moderate 1** | 10.93 | **Moderate 1** | 14.21 | Indicator 7 | 12.51 |
| Moderate 2 | 9.04 | **Moderate 3** | 12.02 | **Factor 2** | 11.83 |
| Indicator 3 | 7.30 | **Severe 4** | 11.05 | **Moderate 1** | 10.64 |
| **Moderate 3** | 5.90 | Indicator 7 | 10.94 | **Severe 4** | 9.84 |
| Indicator 7 | 5.72 | Indicator 3 | 7.09 | **Moderate 8** | 2.12 |
| Moderate 6 | 4.86 | Moderate 6 | 2.34 | **Factor 5** | -0.65 |
| **Moderate 8** | 1.91 | **Moderate 8** | 1.82 | Indicator 3 | -0.82 |
| Indicator 9 | -2.34 | **Factor 5** | -4.38 | **Factor 3** | -4.43 |
| Indicator 1 | -2.76 | **Moderate 5** | -5.93 | **Moderate 5** | -5.72 |
| **Moderate 5** | -4.89 | **Factor 3** | -7.95 | Moderate 6 | -6.14 |
| **Factor 5** | -4.96 | Indicator 1 | -9.20 | Indicator 1 | -19.44 |
| **Factor 3** | -6.32 | Indicator 9 | -9.44 | Indicator 9 | -21.41 |
| Moderate 9 | -11.29 | Moderate 9 | -23.81 | Moderate 9 | -31.48 |
| *AUC of mean predictions (95% CI): 0.797(0.777, 0.818)* | | *AUC of mean predictions (95% CI): 0.791(0.770, 0.811)* | | *AUC of mean predictions (95% CI): 0.777(0.757, 0.798)* | |

*mtry:* The number of variables used for each split of a CART in the forest; AUC: Area under the receiver operating characteristic curve; CI: Confidence Interval. Low prevalence predictors with a prevalence < 10% are in bold.

3. eTable 3: AUC (95% CI), and p-value for DeLong’s test for two correlated ROC curves for each variable selection model for mtry = 6.

| Number of ranked features | AUC (95% CI) | *p-*value for DeLong’s test for two correlated ROC curves |
| --- | --- | --- |
| 6 | 0.69 (0.67, 0.71) | <.001 |
| 7 | 0.70 (0.67, 0.72) | <.001 |
| 8 | 0.71 (0.69, 0.74) | <.001 |
| 9 | 0.72 (0.69, 0.74) | <.001 |
| 10 | 0.71 (0.69, 0.74) | <.001 |
| 11 | 0.72 (0.70, 0.75) | <.001 |
| 12 | 0.74 (0.72, 0.76) | <.001 |
| 13 | 0.74 (0.72, 0.77) | <.001 |
| 14 | 0.76 (0.73, 0.78) | <.001 |
| 15 | 0.76 (0.74, 0.78) | <.001 |
| 16 | 0.77 (0.75, 0.79) | 0.010 |
| 17^a^ | 0.77 (0.76, 0.80) | 0.054 |
| 18 | 0.78 (0.77, 0.80) | 0.057 |
| 19 | 0.78 (0.76, 0.80) | 0.078 |
| 20 | 0.79 (0.76, 0.81) | 0.312 |
| 21 | 0.79 (0.77, 0.81) | 0.852 |
| 22 | 0.79 (0.77, 0.81) | 0.869 |
| 23 | 0.79 (0.77, 0.81) | 0.747 |
| 24 | 0.79 (0.77, 0.81) | 0.770 |
| 25 | 0.79 (0.77, 0.81) | 0.856 |
| 26 | 0.79 (0.77, 0.81) | 0.278 |

AUC: Area under the receiver operating characteristic (ROC) curve; CI Confidence Interval

^a^The most parsimonious model with an AUC that did not differ significantly from the full model.

4. eTable 4: AUC (95% CI), and p-value for DeLong’s test for two correlated ROC curves for each variable selection model for mtry =16.

| Number of ranked features | AUC (95% CI) | *p-*value for DeLong’s test for two correlated ROC curves |
| --- | --- | --- |
| 16 | 0.69 (0.67, 0.71) | <.001 |
| 17 | 0.70 (0.67, 0.72) | <.001 |
| 18 | 0.71 (0.69, 0.74) | <.001 |
| 19 | 0.72 (0.69, 0.74) | <.001 |
| 20 | 0.71 (0.69, 0.74) | <.001 |
| 21 | 0.72 (0.70, 0.75) | <.001 |
| 22 | 0.74 (0.72, 0.76) | <.001 |
| 23 | 0.74 (0.72, 0.77) | <.001 |
| 24^a^ | 0.76 (0.73, 0.78) | 0.009 |

AUC: Area under the receiver operating characteristic (ROC) curve; CI Confidence Interval. ^a^ Selected model with the best AUC. All model AUCs differed significantly from the full model.
